# Unequal Access and Evolving Priorities in U.S. Uveitis Clinical Trials: A 23-year Analysis of Regional, Institutional, and Anatomic trends

**DOI:** 10.1038/s41433-025-03854-7

**Published:** 2025-05-16

**Authors:** Jainam Shah, Sachin Pathuri, Carl S. Wilkins, Andrew D. Parsons, Ibrahim Furkan Acar, Elanit Safaei, Sailesh V. Tummala

**Affiliations:** 1 Albert Einstein College of Medicine, Bronx, NY USA; 2 Creighton University School of Medicine, Phoenix, AZ USA; 3 Department of Ophthalmology, New York Eye and Ear Infirmary of Mount Sinai, New York, NY USA; 4 University of California, Los Angeles, CA USA; 5 Institute for Society and Genetics, Human Biology and Society, University of California, Los Angeles, CA USA; 6 Mayo Clinic Alix School of Medicine, Phoenix, AZ USA; 7 Department of Orthopaedic Surgery, Mayo Clinic, Phoenix, AZ USA

**Keywords:** Epidemiology, Education, Outcomes research

## Introduction

Uveitis is a leading cause of preventable blindness among working-age adults, yet remains underrepresented in ophthalmic research [1]. Disparities in trial access exacerbates inequities affecting underserved communities [2]. This study assesses institutional, geographic, and temporal disparities in U.S. uveitis clinical trials from 2000 to 2023.

## Methods

We identified Phase II–IV uveitis trials between 2000 and 2023 from ClinicalTrials.gov. Trials without U.S. sites or multicenter trials lacking site-level data were excluded. Trials were categorized by sponsorship (public, private, unknown), institution (academic, public, private, unknown), geography (South, West, Midwest, Northeast), and anatomy (anterior, intermediate, posterior, uveitic macular edema, or unspecified/general uveitis). Sponsorship and institution were analysed separately; trials may be academic or public but privately sponsored. Enrollment sizes were compared between institutions using median and interquartile range (IQR). Multinomial logistic regression modelled predictors of private sponsorship. Statistical significance was set at *P* < 0.05. Adjusted odds ratios (aORs) with 95% confidence intervals (CIs) were calculated. Analyses were conducted using SPSS v29. This study was IRB-exempt by the Albert Einstein College of Medicine, as it used public, de-identified data and followed STROBE guidelines.

## Results

98 trials were analysed. Most were in the South (38.8%) and West (30.6%), with fewer in the Northeast (16.3%) and Midwest (14.3%) (Table 1). Private sponsorship accounted for 55.1% of trials, followed by public (27.6%) and unknown (17.3%). Posterior uveitis was most studied (41.8%), while 30.6% of trials were unspecified/general.

**Table 1:** Geographic, Sponsorship, Institutional, and Subtype Distribution of U.S. Uveitis Trials (2000–2023).

Category	Subcategory	Trials (n)	Percent (%)
Region	South	38	38.8
	West	30	30.6
	Northeast	16	16.3
	Midwest	14	14.3
Sponsor Type	Private	54	55.1
	Public	27	27.6
	Unknown	17	17.3
Institution Type	Academic	41	41.8
	Private	31	31.6
	Public	20	20.4
	Unknown	6	6.1
Uveitis Subtype	Posterior	41	41.8
	Intermediate	12	12.2
	Anterior	9	9.2
	Uveitic Macular Oedema	6	6.1
	General/Unspecified	30	30.6

Note: Sponsor and institution classifications are not mutually exclusive; academic or public sites may conduct privately funded trials.

Private trials increased from 28.8% (2000–2011) to 58.6% (2012–2023), while public trials declined from 40.9% to 22.4% (*P* = 0.006). Posterior uveitis trials simultaneously rose from 22.7% to 51.7% (*P* = 0.002). Enrollment varied by institution, with private trials enrolling more participants (Median: 92, IQR 65–110) than public (54; 40–74), academic (36; 26–50), and unknown trials (40; 32–68) (*P* = 0.004).

Posterior uveitis (aOR: 3.41; 95% CI: 1.21–9.61; *P* = 0.020) and South/West location (2.76; 1.03–7.40; *P* = 0.044) predicted private sponsorship (Table 2). Trials from 2012 onwards were more likely privately sponsored (2.84; 1.05–7.68; *P* = 0.039).

**Table 2:** Multivariable Predictors of Private Sponsorship and Trial Enrolment Size.

Predictor/Group	Adjusted OR (95% CI)	P value	Median Enrolment (IQR)
Posterior Uveitis	3.41 (1.21–9.61)	0.020	98 (70–115)
South/West Region	2.76 (1.03–7.40)	0.044	88 (62–110)
Public Institution	Reference	–	54 (40–74)
Academic Institution	0.64 (0.18–2.27)	0.490	36 (26–50)
Unknown	0.71 (0.22–2.34)	0.550	40 (32–68)
Year ≥ 2012	2.84 (1.05–7.68)	0.039	84 (60–112)

Note: Regions were grouped as South/West vs. Northeast/Midwest in regression modelling to improve interpretability and reduce category sparsity.

## Discussion

U.S. uveitis trials from 2000–2023 reveal disparities in sponsorship, scale, and disease focus. The surge in posterior uveitis studies following FDA approval of adalimumab in 2016 suggests a growing emphasis on market-driven endpoints [3]. Although these trials offer broader enrollment, their concentration in the South/West and private sponsorship limit access for patients in the Midwest, Northeast, and public systems. Academic studies on neglected subtypes like intermediate uveitis or macular edema, remain underrepresented.

These findings align with concerns that uveitis care and by extension, access to trials remains concentrated in resource-rich centers, with geographic barriers disproportionately affecting underserved populations [4, 5]. 36.7% of Americans live more than 60 min from a uveitis specialist, and such populations have higher rates of poverty and public insurance [4]. Moreover, fewer than 200 uveitis specialists are active nationally, often lacking support staff or institutional infrastructure to run trials [5]. Public investment and academic incentives are necessary to address these inequities and promote equitable innovation in ophthalmology.

## Data Availability

The data supporting this study’s findings are publicly available from ClinicalTrials.gov. Exclusion criteria were applied to the data obtained from ClinicalTrials.gov for this study. The data used for this study’s analysis will be available from the authors upon reasonable request.
